# Stoichiometry of triple-sieve tRNA editing complex ensures fidelity of aminoacyl-tRNA formation

**DOI:** 10.1093/nar/gky1153

**Published:** 2018-11-12

**Authors:** Lin Chen, Akiko Tanimoto, Byung Ran So, Marina Bakhtina, Thomas J Magliery, Vicki H Wysocki, Karin Musier-Forsyth

**Affiliations:** 1 Department of Chemistry and Biochemistry, The Ohio State University, Columbus, OH 43210, USA; 2 Center for RNA Biology, The Ohio State University, Columbus, OH 43210, USA

## Abstract

Aminoacyl-tRNA synthetases catalyze the attachment of cognate amino acids onto tRNAs. To avoid mistranslation, editing mechanisms evolved to maintain tRNA aminoacylation fidelity. For instance, while rejecting the majority of non-cognate amino acids via discrimination in the synthetic active site, prolyl-tRNA synthetase (ProRS) misactivates and mischarges Ala and Cys, which are similar in size to cognate Pro. Ala-tRNA^Pro^ is specifically hydrolyzed by the editing domain of ProRS in *cis*, while YbaK, a free-standing editing domain, clears Cys-tRNA^Pro^ in *trans*. ProXp-ala is another editing domain that clears Ala-tRNA^Pro^ in *trans*. YbaK does not appear to possess tRNA specificity, readily deacylating Cys-tRNA^Cys^*in vitro*. We hypothesize that YbaK binds to ProRS to gain specificity for Cys-tRNA^Pro^ and avoid deacylation of Cys-tRNA^Cys^ in the cell. Here, *in vivo* evidence for ProRS-YbaK interaction was obtained using a split-green fluorescent protein assay. Analytical ultracentrifugation and native mass spectrometry were used to investigate binary and ternary complex formation between ProRS, YbaK, and tRNA^Pro^. Our combined results support the hypothesis that the specificity of YbaK toward Cys-tRNA^Pro^ is determined by the formation of a three-component complex with ProRS and tRNA^Pro^ and establish the stoichiometry of a ‘triple-sieve’ editing complex for the first time.

## INTRODUCTION

Aminoacyl-tRNA synthetases (ARSs) pair cognate amino acids with the corresponding tRNAs to form aminoacyl-tRNAs, which are delivered to the ribosome for protein synthesis via elongation factors (EF-Tu in bacteria and EF-1α in archaea and eukaryotes) (1). This process is achieved in two steps: first, an aminoacyl-adenylate intermediate is formed in which amino acids are activated by ATP; then the activated amino acids are transferred to the 3′ end of tRNAs. ARSs can be divided into two classes based on catalytic domains: the class I ARS active site contains a Rossmann nucleotide-binding fold active site domain, while class II ARSs contain an antiparallel β-sheet active site (2). Due to the structural similarities of amino acids, ARSs are error prone (3). High fidelity amino acid selection by ARSs is essential because an accumulation of mistakes in protein translation can cause protein misfolding and can even lead to cell death (4–7). To maintain the accurate flow of genetic information and cellular homeostasis, some ARSs have evolved proofreading or editing capabilities. Editing of noncognate aminoacyl-adenylates is known as ‘pre-transfer’ editing and deacylation of mischarged aminoacyl-tRNAs is called ‘post-transfer’ editing (8). Misactivated amino acids can be edited in the synthetic active site of some ARSs, such as class II seryl-tRNA synthetase (SerRS) (9), whereas a distinct active site, the connective peptide 1 (CP1) domain, is inserted into the Rossmann-fold domain and edits tRNAs mischarged by three class I ARSs: isoleucyl-tRNA synthetase, valyl-tRNA synthetase, and leucyl-tRNA synthetase (10). A dual active site model (later termed the ‘double-sieve’ model (11,12)) wherein distinct active sites are responsible for aminoacylation (coarse sieve) and post-transfer editing (fine sieve) was first proposed by Fersht and Kaethner (13). Post-transfer editing also takes place within distinct domains in the case of class II synthetases, such as alanyl-tRNA synthetase (AlaRS) (7), threonyl-tRNA synthetase (ThrRS) (14), phenylalanyl-tRNA synthetase (15) and prolyl-tRNA synthetase (ProRS) (16). Additionally, *trans*-editing factors, which are single-domain proteins homologous to the editing domains of AlaRS (17), ThrRS (18) and ProRS (17,19,20) are encoded in the genomes of many organisms.

ProRS can mischarge Ala and Cys onto tRNA^Pro^ (16,21). In many bacteria including *Escherichia coli*, a ‘triple-sieve’ mechanism has been proposed to ensure proline codon translation fidelity (20). While rejecting the majority of non-cognate amino acids that are too large to bind in the active site pocket (coarse sieve), ProRS uses a distinct insertion (INS) (second sieve) domain to deacylate Ala-tRNA^Pro^; however, it is not active against Cys-tRNA^Pro^. Instead, YbaK, a free-standing protein that is homologous to the INS domain, is the third sieve that deacylates Cys-tRNA^Pro^ in *trans* (20,22). An alternative triple-sieve mechanism of editing has been described in *Caulobacter crescentus*, which exclusively relies on *trans*-editing domains for post-transfer editing (19). *Caulobacter crescentus* ProRS does not possess an INS domain; instead, ProXp-ala, an independent editing protein, deacylates Ala-tRNA^Pro^. Similar to *E. coli*, the third sieve of tRNA^Pro^ editing is YbaK, which clears Cys-tRNA^Pro^. YbaK is a general Cys-tRNA deacylase (22). In previous studies, it was hypothesized that YbaK binds to ProRS to gain specificity for Cys-tRNA^Pro^ and avoid deacylation of Cys-tRNA^Cys^*in vivo* (23). Moreover, the fact that EF-Tu can protect aminoacyl-tRNA from deacylation by YbaK suggests hydrolysis of mischarged tRNA by YbaK occurs before ProRS release (23). Oxidative crosslinking results also suggested the formation of ProRS/YbaK binary and ProRS/tRNA^Pro^/YbaK ternary complexes (23). However, the ternary complex has been difficult to characterize due to its apparently transient nature, and the stoichiometry of the complex is unknown.

In this study, a split-green fluorescent protein (GFP) assay was used to probe the interaction between *E. coli* ProRS and YbaK *in vivo*. Analytical ultracentrifugation (AUC) and native nanoelectrospray ionization (nESI) coupled to ion mobility-mass spectrometry (IM-MS) were used to investigate binary and ternary complex formation between ProRS, tRNA^Pro^ and YbaK. Using native MS, we determined the stoichiometry of all two and three-component complexes. Additionally, a MS competition binding study, performed by using a high-resolution extended mass range Orbitrap™ (EMR) spectrometer, confirmed the specificity of tRNA binding for ProRS and ProXp-ala, which is in contrast to the relatively nonspecific binding of tRNAs to YbaK. These data support the triple-sieve mechanism of tRNA^Pro^ editing and establish the editing complex stoichiometry for the first time.

## MATERIALS AND METHODS

### Protein preparation

Unless otherwise indicated, all buffers and reagents were from Sigma-Aldrich. Overexpression of *C. crescentus* ProRS, YbaK, ProXp-ala and *E. coli* ProRS, CysRS, YbaK and EF-Tu, as well as subsequent purification using the His-select^®^ nickel affinity resin were performed as previously described (19,20,24–26). Biotinylated thrombin (Novagen) was used to cleave after the 6-His tag, generating non-tagged *C. crescentus* ProRS, YbaK and ProXp-ala. Streptavidin-agarose resin (Thermo Scientific) was applied to remove thrombin. Purified proteins were buffer exchanged into 200 mM ammonium acetate buffer adjusted to pH 7.5 with ammonia, and kept on ice until MS analysis, with MS analysis typically performed on the same day. Protein concentrations were determined either by the Pierce™ BCA Protein Assay Kit (Thermo Fisher Scientific) or by a UV-Vis NanoDrop Spectrophotometer (Thermo Fisher Scientific) using protein extinction coefficients determined using the ProtParam tool in ExPasy (http://web.expasy.org/protparam/) (27). Proteins were estimated to be >95% pure by SDS PAGE and visualization by Coomassie Blue staining and high protein purity (>98%) was confirmed by mass spectrometry.

### tRNA preparation

*Escherichia coli* tRNA^Pro^, tRNA^Cys^, and tRNA^Ala^ were prepared by *in vitro* transcription using T7 RNA polymerase as described previously (16). The plasmid encoding tRNA^Pro^ contains a hammerhead ribozyme 5′ of the tRNA gene to ensure high yields of this C1-containing tRNA transcript (28). Thus, tRNA^Pro^ has a 5′-monophosphate end, whereas the other two tRNAs have 5′-triphosphate ends. The tRNA^Ala^ plasmid used for these studies encodes a single-base change in the anticodon (G35A), which does not affect aminoacylation by AlaRS or editing by the single-domain *trans*-editing proteins investigated here. Before experiments, tRNA was refolded by heating at 80°C for 2 min, 60°C for 2 min, followed by addition of 10 mM Mg(OAc)_2_ or MgCl_2_ and cooling to room temperature for 3 min. Samples used in native MS were prepared in 200 mM ammonium acetate buffer, pH 7.5, and after refolding were kept on ice until MS analysis.

### Split-GFP assembly assay

Construction of plasmids and expression for split-GFP reassembly were performed according to published protocols (29). pET11a-NGFP constructs encoding *E. coli* ProRS and *E. coli* YbaK sequences (NGFP-ProRS and NGFP-YbaK) and a pMRBAD-CGFP construct encoding *E. coli* ProRS (CGFP-ProRS) were prepared using standard PCR cloning strategies. Plasmid sequences were confirmed by DNA sequencing. The plasmids encoding pET11a-Z-NGFP (NGFP-zipper), pMRBAD-Z-CGFP (CGFP-zipper), and pMRBAD-link-CGFP (CGFP-linker) were constructed as described (30). *E. coli* BL21 (DE3) cells were cotransformed with the following pairs of plasmids: (I) NGFP-zipper and CGFP-zipper; (II) NGFP-ProRS and CGFP-ProRS; (III) NGFP-YbaK and CGFP-YbaK; (IV) NGFP-YbaK and CGFP-ProRS; (V) NGFP-YbaK and CGFP-linker; (VI) NGFP-ProRS and CGFP-linker. Single colonies were selected and grown in LB media containing 100 μg/ml ampicillin and 35 μg/ml kanamycin at 37°C overnight. Overnight cultures were diluted 1000-fold and 10-μl aliquots were spread on 2× YT agar plates containing 100 μg/ml ampicillin, 35 μg/ml kanamycin, 10 μM IPTG and 0.2% arabinose. The plates were incubated at room temperature for 36 h. Plate images were obtained with a Typhoon FLA 9500 fluorescence scanner (GE Healthcare) equipped with 473 nm blue LD laser and 575 nm LPG filter.

### Analytical ultracentrifugation

Analytical ultracentrifugation (AUC) experiments were conducted using an Optima XL-I ultracentrifuge (Beckman Coulter) with an An50 Ti rotor and standard double-sector Epon centerpieces. Sedimentation velocity experiments were performed at 20°C at a rotor speed of 50000 rpm in a buffer containing 50 mM HEPES (pH 6.8), 30 mM KCl and 1 mM MgCl_2_. YbaK was labeled on surface Lys residues with Alexa Fluor 680 succinimidyl ester (AF680) using a protein labeling kit (Molecular Probes) according to the manufacturer's protocol. Absorption at 280 nm and 679 nm was measured and protein concentration was determined using the following equation [AF680-YbaK] = (Abs_280_ – 0.05*Abs_679_)/7450, where 7450 is the extinction coefficient of YbaK at 280 nm. Concentration of incorporated label was determined as [AF680] = (Abs_679_/184 000), where 184 000 is the extinction coefficient of AF680 at 679 nm. Labeling stoichiometry was kept at <1 probe per YbaK molecule. Protein and nucleic acid samples were equilibrated with the AUC buffer by elution through a Superdex200 Increase 10/300 column (GE Healthcare). The following concentrations were used: 10 μM tRNA^Pro^, 10 μM ProRS, 2.5 μM YbaK. Data were collected by monitoring absorbance at either 260, 280 or 679 nm. AUC data were analyzed using SEDFIT (https://sedfitsedphat.nibib.nih.gov/software/default.aspx) (31).

### Native mass spectrometry

A modified Synapt G2 HDMS (Waters, Manchester, UK) quadrupole/ion mobility/time-of-flight instrument was used to perform native MS. This instrument has a customized surface-induced dissociation (SID) device installed between the trap T-wave and the IM cell. The samples were introduced into the instrument by nanospray by using a borosilica capillary, which was pulled in-house on a P-97 micropipette puller (Sutter Instrument, Hercules, CA, USA). Voltages of 1.0–1.5 kV were applied with a platinum wire inserted into the sample solution at the back of the capillary. Typical instrument settings for MS experiments were sampling cone 20 V, source offset 2 V, source at room temperature, desolvation temperature 260°C, trap DC entrance at 0 V, and bias DC at 45 V (75–145 V for SID voltage corresponding to 30 V-100 V), trap gas flow at 4 ml/min, helium cell gas flow at 120 ml/min, trap wave velocity at 160 m/s, trap wave height at 4 V, IMS entrance at 10 V, helium cell DC at 25 V, helium exit at –5 V, IMS DC bias at 3 V, IMS exit at 0 V, IMS gas flow at 60 ml/min, IMS wave velocity at 300 m/s, IMS wave height at 20 V, transfer DC entrance at 0 V, transfer DC exit at 5 V, transfer wave velocity at 45 m/s, and transfer wave height at 0.5 V. The final spray solution for the sample contained 200 mM ammonium acetate, pH 7.5 and 0.5 mM Mg^2+^ in the experiments with tRNAs. For individual components, proteins and tRNAs were sprayed at a concentration of 1 μM. ProRS and tRNA^Pro^ were mixed at a final concentration of 10 μM each to observe the binary complex. YbaK and tRNA^Pro^ were mixed at a concentration of 15 and 10 μM, respectively, to observe the binary complex. ProRS, YbaK and tRNA^Pro^ were mixed at final concentrations of 10, 30 and 20 μM, respectively, to observe the ternary complex. Ternary complex is used throughout the manuscript to refer to the number of unique components and not the stoichiometry.

### MS competition studies

Competition studies were performed on an Exactive EMR Orbitrap MS (Thermo Scientific). The samples were sprayed by nanoESI in a borosilica capillary, as described above. Typical instrument settings were sample spray voltage at 1.0–1.5 kV, in-source dissociation at 60 V, higher energy collisional dissociation (HCD) voltage at 90 V, desolvation temperature at 250°C, source offset voltage at 20 V, S-lens RF level at 150, injection flatapole DC at 8 V, inter flatapole lens at 8, bent flatapole DC at 6 V, transfer multipole DC tuning offset at 0 V, C-trap entrance lens tuning offset at 5 V, high vacuum pressure at 3.5e^−9^ mbar, and resolving power at 8750 (FWHM at *m/z* 200). For the competition studies, tRNA samples were buffer exchanged into 200 mM ammonium acetate, pH 7.5, to remove excess salt, using Micro Bio-spin 6 columns (BioRad). ProXp-ala (5 μM) was first mixed with 1 μM tRNA^Cys^, tRNA^Ala^ or tRNA^Pro^ in 1 mM Mg(OAc)_2_ and analyzed separately to observe the binary complexes of ProXp-ala/tRNA^Cys^, ProXp-ala/tRNA^Ala^ and ProXp-ala/tRNA^Pro^. ProXp-ala (5 μM) was then mixed with all three tRNAs at a final concentration of 0.33 μM of each tRNA and analyzed. Competition assays for YbaK were carried out similarly. To validate the binary complexes of ProXp-ala/tRNA and YbaK/tRNA, monoisotopic distribution graphs were generated from the chemical formula of the binary complexes using an online tool in ChemCalc (http://www.chemcalc.org/) (32).

The binary complexes of ProRS with individual tRNAs were also analyzed. ProRS (1 μM) was first mixed with either 10 μM tRNA^Cys^, tRNA^Ala^ or tRNA^Pro^. Next, all three tRNAs (3.3 μM each of tRNA^Cys^, tRNA^Ala^ and tRNA^Pro^) were mixed to compete for binding to ProRS (1 μM). Similar competition studies for formation of the ternary complex of ProRS/YbaK/tRNA were also conducted. A mixture of 1 μM ProRS, 20 μM YbaK and 10 μM tRNA^Cys^, tRNA^Ala^ or tRNA^Pro^ were first analyzed to observe the individual ternary complexes. Next, all three tRNAs (3.3 μM each tRNA^Cys^, tRNA^Ala^ and tRNA^Pro^) were mixed to compete for binding to ProRS (1 μM) and YbaK (20 μM).

While the binary complexes of ProXp-ala/tRNA and YbaK/tRNA and their corresponding salt- adduct peaks are well resolved in the *m/z* region due to their smaller masses, binary and ternary complexes of ProRS/tRNA and ProRS/YbaK/tRNA are larger in mass and appear in the higher *m/z* region. Due to the overlapping salt-adduct peaks, instead of generating monoisotopic distribution graphs to validate the MS peaks in these cases, a Gaussian function was used to simulate the predicted MS peaks of the binary and ternary complexes, as described in supplementary information (Supplementary Table S1).

### EF-Tu competition assays

Preparation of [^35^S]-Cys-tRNA^Pro^ and [^35^S]-Cys-tRNA^Cys^ substrates were performed as described (20). *Escherichia coli* EF-Tu was activated in 50 mM Na·HEPES (pH 7.2), 1 mM DTT, 68 mM KCl, 6.7 mM MgCl_2_, 2.5 mM phosphoenolpyruvate, 0.5 mM GTP and 30 μg/ml pyruvate kinase at 37°C for 1 h before use (33). Deacylation assays were carried at 37°C as previously described (33) using the following protein and substrate concentrations: 0.1 μM YbaK, 0.5 μM ARS, 0.5 μM activated EF-Tu, 0.5 μM Cys-tRNA^Pro^.

## RESULTS

### Split-GFP assay

**Figure 1.**
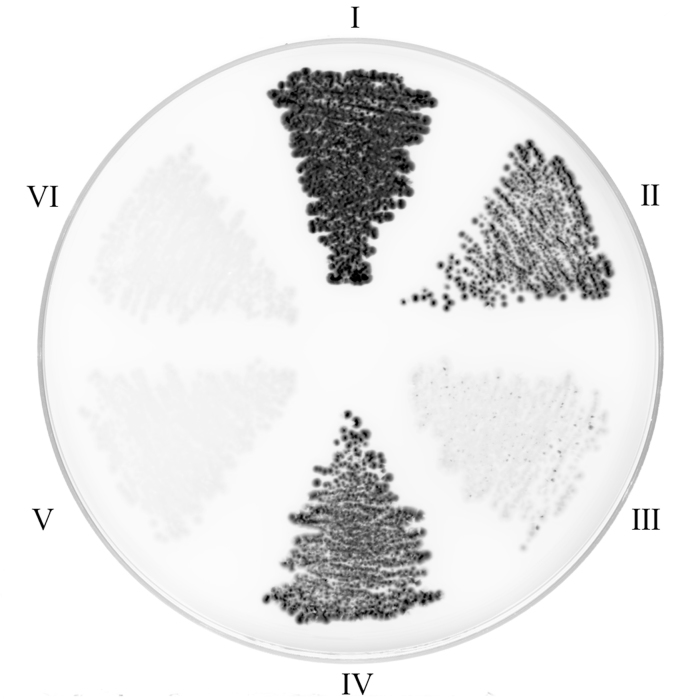
Probing ProRS-YbaK interaction in cells using split-GFP reassembly assay. (I) NGFP-zipper and CGFP-zipper; (II) NGFP-ProRS and CGFP-ProRS; (III) NGFP-YbaK and CGFP-YbaK; (IV) NGFP-YbaK and CGFP-ProRS; (V) NGFP-YbaK and CGFP-linker; (VI) NGFP-ProRS and CGFP-linker.

To probe the interaction between *E. coli* ProRS and YbaK, a split GFP reassembly method was carried out. Previously, the binding dissociation constant of *E. coli* ProRS with fluorophore-labeled YbaK was reported to be ∼550 nM and this binding affinity was reported to strengthen (*K*_d_ = 45 nM) in the presence of tRNA^Pro^ (23). However, a shortcoming of this assay was the inability to distinguish between increased affinity to ProRS due to the presence of tRNA or formation of a high-affinity tRNA-YbaK binary complex that binds to ProRS. Even though crosslinking experiments supported the formation of the ternary complex *in vitro* (23), the ternary complex has not been observed in the cell or in the absence of crosslinking *in vitro*. The split-GFP assay is a powerful and direct approach to investigate protein-protein interactions (29,34). Pairs of proteins of interest are fused to one of the two split-GFP molecules (NGFP: residues 1–157; CGFP: residues 158–238), and their association will result in cellular fluorescence (29). As a positive control, a known coiled-coil leucine-zipper domain protein-protein interaction was tested. As expected, a strong fluorescence signal was observed (Figure 1, panel I). Panel II shows a strong fluorescence emission upon co-expression of NGFP-ProRS and CGFP-ProRS, which is consistent with the known homodimerization of class II synthetases (2). Interestingly, NGFP-YbaK interacted with CGFP-ProRS (panel IV), whereas YbaK did not homodimerize (panel III). YbaK and ProRS did not interact with a short peptide linker (negative controls, panel V and VI). These data show that ProRS and YbaK form a complex *in vivo*, but the experiments do not directly probe formation of a ternary complex with tRNA^Pro^.

### Analytical ultracentrifugation studies

**Figure 2.**
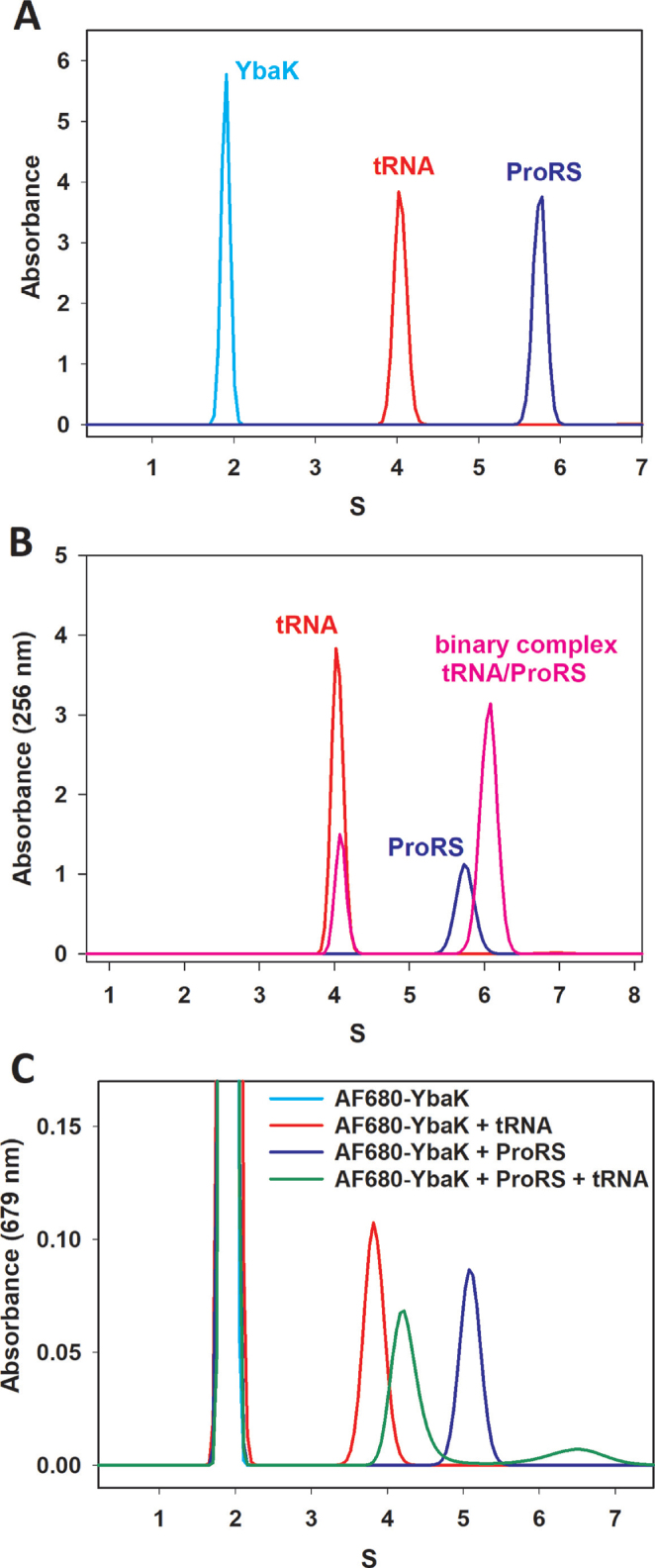
Sedimentation coefficient distribution of *C. crescentus* ProRS, *C. crescentus* YbaK, *E. coli* tRNA^Pro^ and their complexes. (**A**) AUC results of individual species. ProRS (blue) absorbance at 280 nM, AF680-YbaK (cyan) absorbance at 679 nM, and tRNA^Pro^ (red) absorbance at 256 nM. (**B**) ProRS alone (blue), tRNA^Pro^ (red), and ProRS mixed with tRNA^Pro^ (pink). (**C**) AF680-YbaK (cyan), AF680-YbaK mixed with ProRS (blue), AF680-YbaK mixed with tRNA^Pro^ (red) and AF680-YbaK mixed with ProRS and tRNA^Pro^ (green).

To evaluate whether YbaK, ProRS, and tRNA^Pro^ form a ternary complex, we first performed a series of sedimentation velocity (SV) AUC experiments with each molecule separately, monitoring absorbance at 679 nm (AF680-YbaK), 280 nm (ProRS), and 256 nm (tRNA). Figure 2A shows that each sample was monodisperse and all molecules were very well behaved with no indication of aggregation or degradation. *C. crescentus* ProRS had a sedimentation coefficient of 5.78 S, which suggests a MW of 96.9 kDa. Since the predicted MW of a *C. crescentus* ProRS monomer is 49.5 kDa, the SV results are consistent with the expectation that ProRS exists as a dimer in solution. Molecular weights derived from SV results for *C. crescentus* YbaK and *E. coli* tRNA^Pro^ are 16.3 and 25.7 kDa, respectively, indicating that YbaK and tRNA are monomers in solution. Analysis of data collected at different wavelengths gave similar sedimentation coefficients (Supplementary Table S2).

To study tRNA^Pro^ interaction with ProRS, we first collected SV data by monitoring absorbance at 256 nm. Formation of the ProRS/tRNA^Pro^ binary complex produced a shift of the ProRS peak (from 5.75 S to 6.06 S) (Figure 2B). Upon formation of the binary complex, the intensity of the signal from free tRNA^Pro^ decreased and this decrease was accompanied by an increase of the binary complex signal at 6.06 S. Fluorescently-labeled AF680-YbaK was used to detect the YbaK/tRNA^Pro^ and YbaK/ProRS binary complexes. The free YbaK peak at ∼2 S shifted to a species that absorbed at 679 nm and had a sedimentation coefficient of 3.83 S in the presence of tRNA^Pro^ (Figure 2C). The interaction of YbaK and ProRS was observed as a peak with a sedimentation coefficient of 5.09 S (Figure 2C). When ProRS, tRNA^Pro^, and AF680-YbaK were mixed, two new peaks were observed. We presume that the larger peak at 4.3 S is the YbaK/tRNA binary complex, whereas a minor peak at 6.4 S reflects the ProRS/tRNA/YbaK ternary complex (Figure 2C). Surprisingly, the S value for the ProRS/YbaK complex (5.09) was lower than the value for ProRS alone (5.9). This may be explained by a conformational change upon binding that resulted in an increase in the frictional coefficient and a decrease in the sedimentation coefficient of the complex compared to ProRS alone. In addition, the *S* value for the tRNA/YbaK binary complex in the absence of ProRS (3.83) shifted to 4.3*S* in the ternary complex. This may be due to the dynamic equilibrium between binary and ternary complexes, which influenced their hydrodynamic properties.

### Native mass spectrometry

To reveal the stoichiometry of the binary and ternary complexes of ProRS, YbaK and tRNA, native MS was performed. Since MS analysis requires the use of volatile buffers, the enzymatic activity of ProRS and YbaK was tested in ammonium acetate buffer using aminoacylation and deacylation assays, respectively. These assays showed that the enzyme activities in 200 mM ammonium acetate, pH 7.5, are comparable with those measured using conventional non-volatile buffers such HEPES (Supplementary Figure S1).

The MS peak envelopes of tRNAs measured in native conditions (200 mM ammonium acetate, 0.5 mM Mg^2+^) on the Exactive EMR (Orbitrap) were not as broad as those on the Synapt G2 because of a higher efficiency of declustering. The observed mass was higher than the theoretical mass (data not shown) because of adducting with water, ammonium, and magnesium ions. The two most abundant peaks in the cluster corresponded to addition of 78 or 102 Da while the highest m/z peaks in the cluster correspond to addition of up to 186 Da (combinations of H_2_O, NH_4_^+^ and Mg^2+^). Supplementary Figure S2 shows native MS analysis of the three proteins alone (A–C) and three tRNA species (D–F). As expected based on the AUC data, we observed exclusively monomeric ProXp-ala and YbaK and predominantly dimeric ProRS. The tRNAs were also exclusively monomeric, as expected.

**Figure 3.**
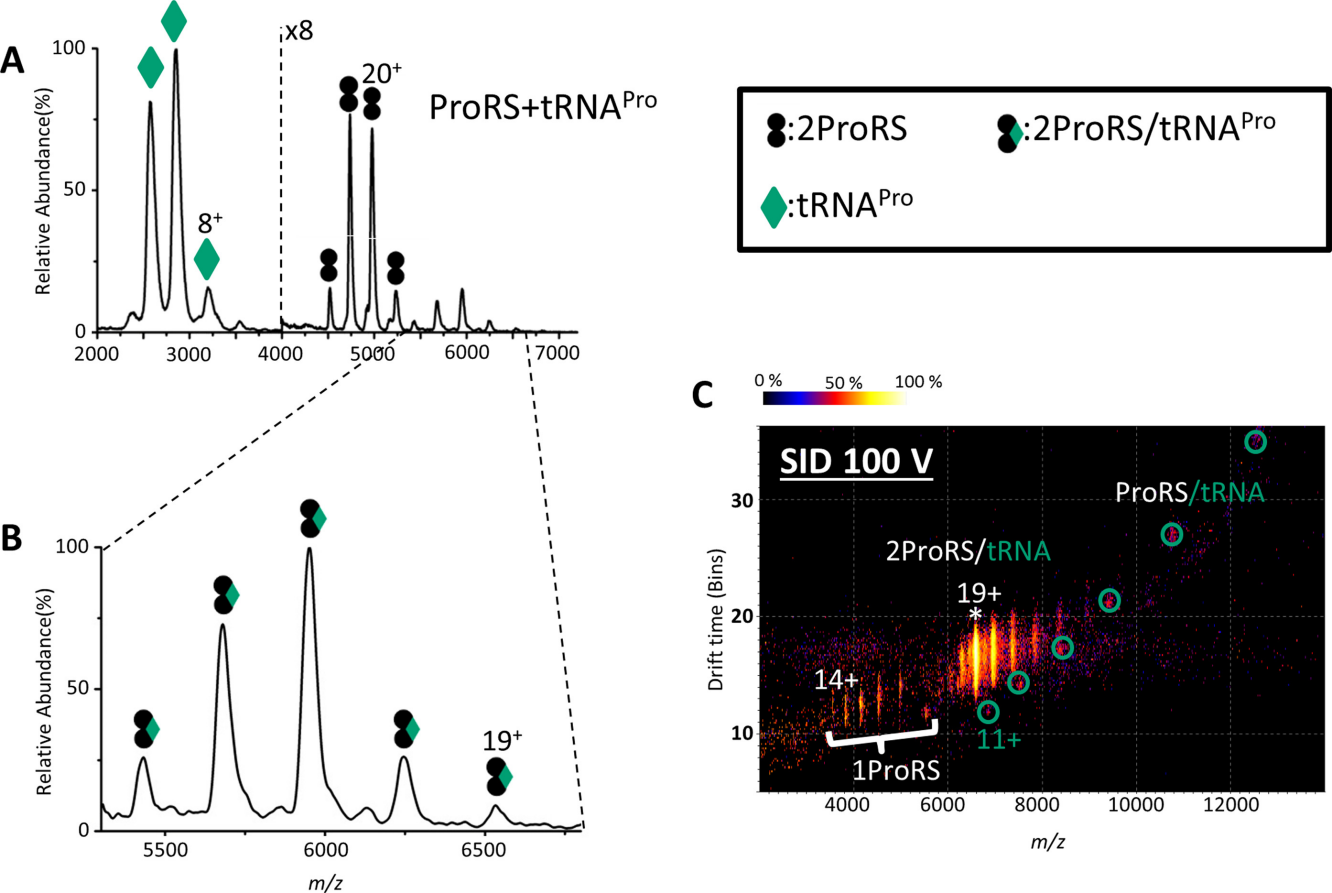
Native mass spectra and ion mobiligram of the binary complex of ProRS/tRNA^Pro^. (**A**) Mass spectrum of a mixture of ProRS and tRNA^Pro^. (**B**) Zoom in of the high *m/z* region (5300–6800 m/z) of (A) showing the complex of two ProRS with one tRNA^Pro^; the observed mass of the binary complex is 124.5 kDa whereas the theoretical mass is 123.8 kDa. (**C**) Ion mobiligram illustrating products after SID (100 V; 1900 eV) of the binary complex. * represents the precursor peak that was isolated for SID (adjacent peaks are from charge stripping and reflect different charged states). ProRS monomer peaks are indicated and ProRS/tRNA complex peaks are circled.

Upon mixing ProRS and tRNA^Pro^ over a range of ratios, a series of new peaks corresponding to two ProRS bound to one tRNA^Pro^ were observed, in addition to free ProRS dimer and tRNA monomer (Figure 3A, B). This binary complex was confirmed by SID, which dissociates the complex by collision against a fluorocarbon-coated gold surface (35–37). The complex dissociated into ProRS monomer and ProRS bound to one tRNA^Pro^ molecule (ProRS/tRNA^Pro^), confirming the 2:1 ProRS/tRNA^Pro^ complex. The ion mobility drift time spectrum is also consistent with this conclusion, showing clear separation of dissociated ProRS monomer and ProRS/tRNA^Pro^ binary complex (Figure 3C).

**Figure 4.**
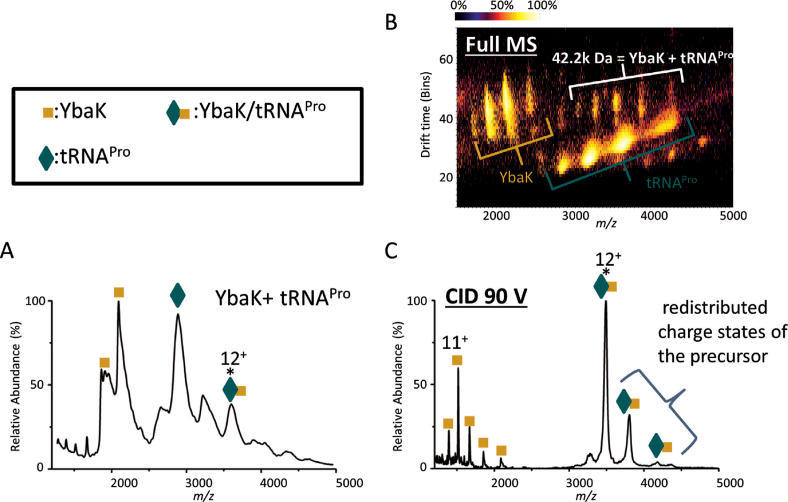
Native mass spectra and ion mobiligram of the binary complex of YbaK/tRNA^Pro^. MS spectra of (**A**) a mixture of YbaK and tRNA^Pro^. The observed mass of the binary complex is 42.2 kDa, whereas the theoretical mass is 41.4 kDa. (**B**) Ion mobility of A with CID 70V applied to ‘clean’ spectra, but no m/z selection, separating the 42.2 kDa peaks from tRNA^Pro^. (**C**) CID 90 V (1080 eV) on charge state 12+ of the binary complex. * represents the precursor peak that was picked for CID.

**Figure 5.**
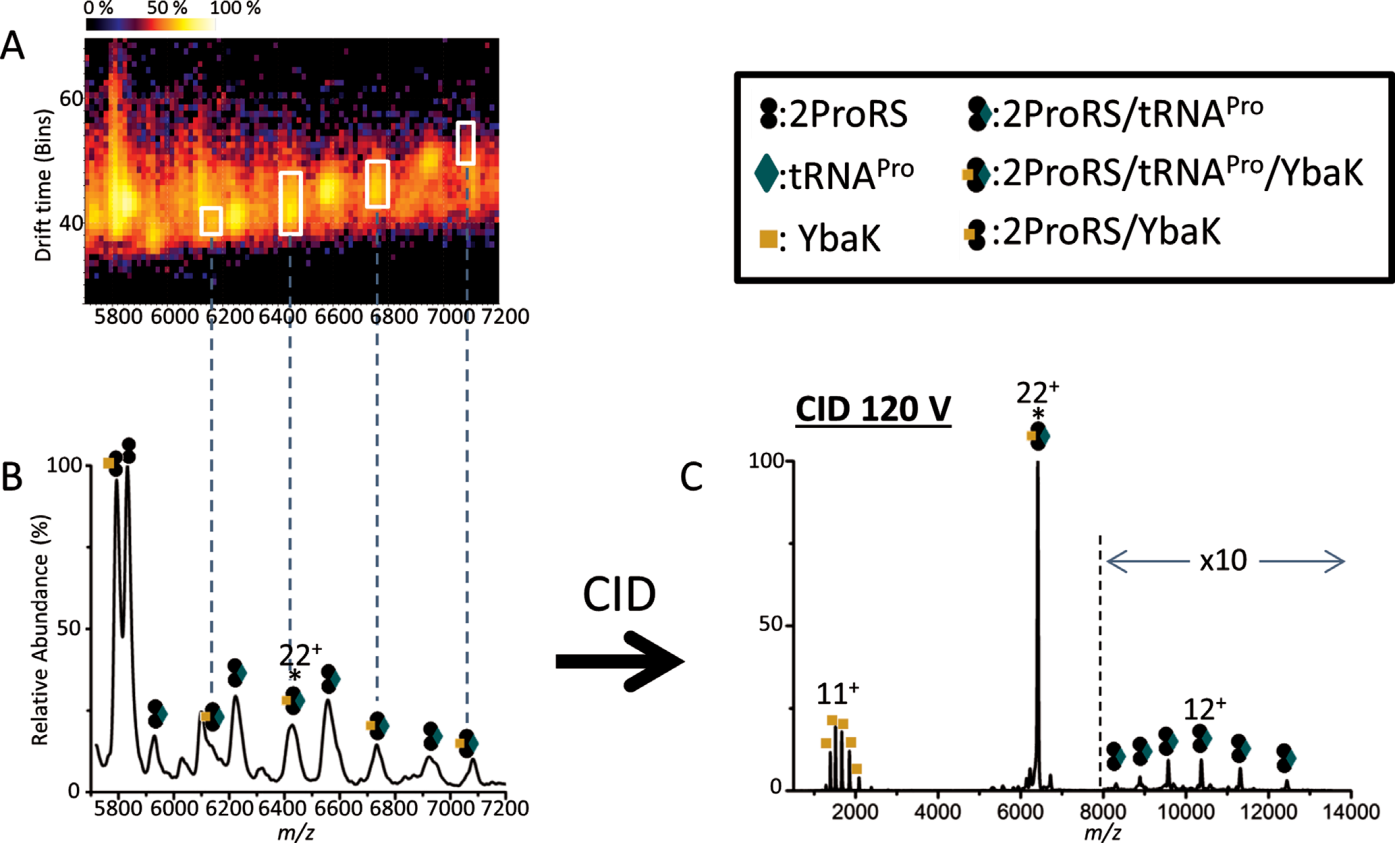
Native MS analysis of the ProRS/tRNA^Pro^/YbaK ternary complex. (**A**) Ion mobiligram with quad profile at *m/z* = 7000 of the ProRS/tRNA^Pro^/YbaK ternary complex. (**B**) MS spectrum of A. The observed mass of the ternary complex is 141 kDa whereas the theoretical mass is 140 kDa. (**C**) CID 2640 eV of the ternary complex (22^+^); adjacent peaks to the right of the selected charge state correspond to loss of 1 and 2 positive charges, respectively (11^+^ and 10^+^ precursor) * represents the precursor peak that was picked for CID.

We next investigated the YbaK/tRNA^Pro^ binary complex. In addition to observing free YbaK and free tRNA^Pro^, a new peak was observed representing the binary complex (Figure 4A). The ion mobiligram, confirmed that a binary complex of 1 YbaK and 1 tRNA is present in addition to YbaK and tRNA monomers (Figure 4B). When the 12+ charge state of the heterodimeric YbaK/tRNA complex was mass-selected and fragmented by CID, a YbaK monomer was observed, confirming that YbaK is contained in the selected peak (Figure 4C). However, dissociated tRNA was not observed. We speculate that charges on tRNA were depleted by unfolded YbaK upon their dissociation and become undetectable by MS. The dissociation behavior of protein-RNA complexes has not been extensively investigated by MS, and further studies are required. Although dissociated tRNA was not observed, in addition to the remaining selected 12+ precursor peaks, charge-stripped peaks 11+ and 10+ were also present, allowing confirmation of the precursor mass as equal to the sum of one YbaK and one tRNA. Finally, analysis of a mixture of all three components, ProRS, YbaK, and tRNA^Pro^, revealed the presence of complex peaks that were consistent with a ProRS:tRNA^Pro^:YbaK stoichiometry of 2:1:1 (Figure 5A and B). When the 22+ charge state of the ternary complex was fragmented by CID, a 2ProRS/tRNA^Pro^ complex and a YbaK monomer dissociated from the complex, confirming that the three-component complex consisted of two ProRS, one tRNA, and one YbaK (Figure 5C).

### MS competition-binding study

**Figure 6.**
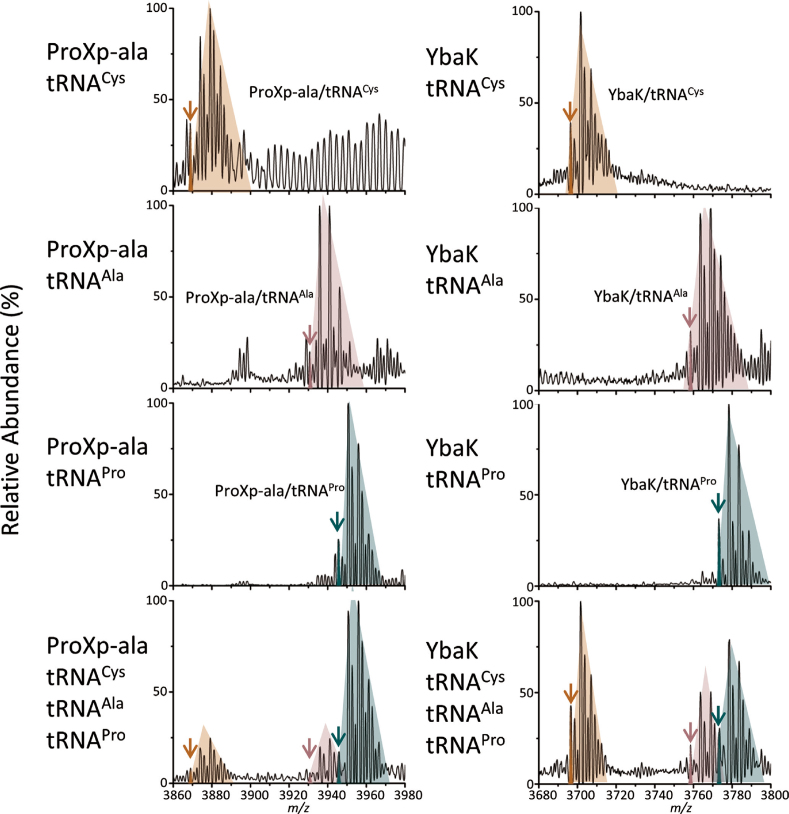
Competition studies of ProXp-ala and YbaK with different tRNAs. Native mass spectra of ProXp-ala (left) and YbaK (right) with individual tRNAs and a mixture of tRNA^Pro^, tRNA^Ala^ and tRNA^Cys^. Theoretical monoisotopic distribution plots for complexes (11+) of protein/tRNA^Cys^ (orange), tRNA^Ala^ (pink), and tRNA^Pro^ (green) are overlaid on the experimental data (black). Peaks, which match with the theoretical plots are indicated with arrows. The peaks in the shaded colored region correspond to complexes (11+) of protein/tRNA^Cys^ (orange), tRNA^Ala^ (pink), and tRNA^Pro^ (green) adducted by Mg^2+^, NH^4+^ and water.

**Figure 7.**
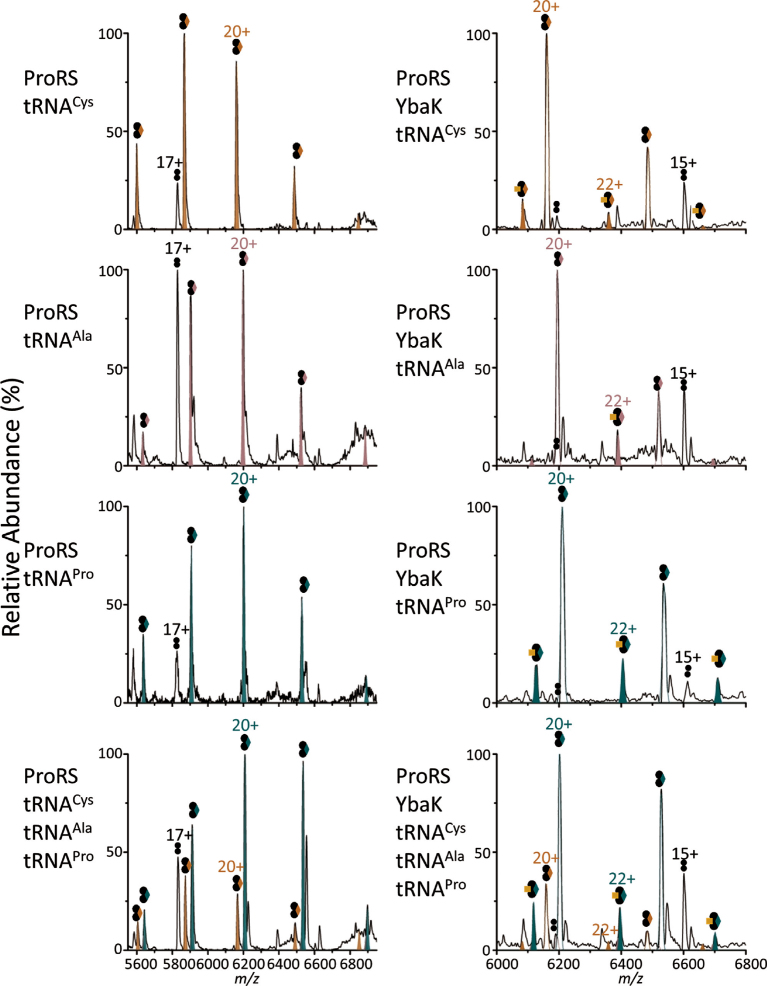
Competition studies of ProRS, and ProRS/YbaK with different tRNAs. Left: Native mass spectra of ProRS with individual tRNAs and a mixture of tRNA^Pro^, tRNA^Ala^ and tRNA^Cys^. Gaussian simulation plots for complexes of ProRS/ tRNA^cys^ (orange), tRNA^ala^ (pink), and tRNA^Pro^ (green) are filled in and overlaid on the experimental data (black) Right: Native MS of ProRS and YbaK with individual tRNAs and a mixture of tRNA^Pro^, tRNA^Ala^, and tRNA^Cys^. Simulated peaks for ternary complexes with tRNA^Cys^ (orange), tRNA^Ala^ (pink), and tRNA^Pro^ (green) are filled in, and plots for binary complexes of ProRS with tRNA^Cys^ (orange), tRNA^Ala^ (pink), and tRNA^Pro^ (green) are shown in solid outline overlaid on the experimental data (black). Representations of symbols are indicated in Figure 5.

A competition study was performed to investigate the binding specificity of ProRS, ProXp-ala, and YbaK towards cognate tRNA^Pro^. Samples were analyzed on an Exactive Plus (EMR) Orbitrap MS. In this analysis, complex formation was probed in the presence of individual tRNA species or for equimolar mixtures of three tRNA species. An advantage of this approach is that complex formation for each protein with different tRNAs can be directly visualized and compared in a single spray without the use of any artificial tags. The use of a high-resolution mass spectrometer was essential to resolve the small mass differences and distinguish complexes with different tRNAs. The Exactive EMR is an ideal mass analyzer for this study because of its high transmission efficiency for high masses, sensitivity, and resolution. To test the concept of this competition study using native MS, we first performed the analysis on *C. crescentus* ProXp-ala, which is known to bind cognate tRNA^Pro^ specifically (19,38) and is therefore predicted to form a complex with tRNA^Pro^ preferentially over tRNA^Ala^ or tRNA^Cys^. Consistent with this prediction, greater complex formation was observed between ProXp-ala and tRNA^Pro^ relative to the non-cognate tRNAs (a ratio of roughly 4:1:1 for tRNA^Pro^:tRNA^Cys^:tRNA^Ala^ is illustrated in the experimental data of Figure 6 (left bottom panel). Similarly, when ProRS was mixed with different tRNAs and analyzed on the EMR, ProRS formed a higher amount of complex with tRNA^Pro^ compared to the two non-cognate tRNAs, indicating that ProRS has specificity toward its cognate tRNA, as expected (Figure 7, left panels). In contrast, YbaK showed no significant preference for binding to a specific tRNA (Figure 6, right panels). However, when both ProRS and YbaK were mixed with different tRNAs, a ternary complex consisting of a ProRS dimer, YbaK and one tRNA^Pro^ formed to a significantly greater extent than complexes containing tRNA^Ala^ or tRNA^Cys^ (Figure 7, right panels). This result supports the idea that YbaK alone cannot recognize cognate tRNA^Pro^; however, it can recognize tRNA^Pro^ via interaction with ProRS by forming a ternary complex.

### Deacylation assays

**Figure 8.**
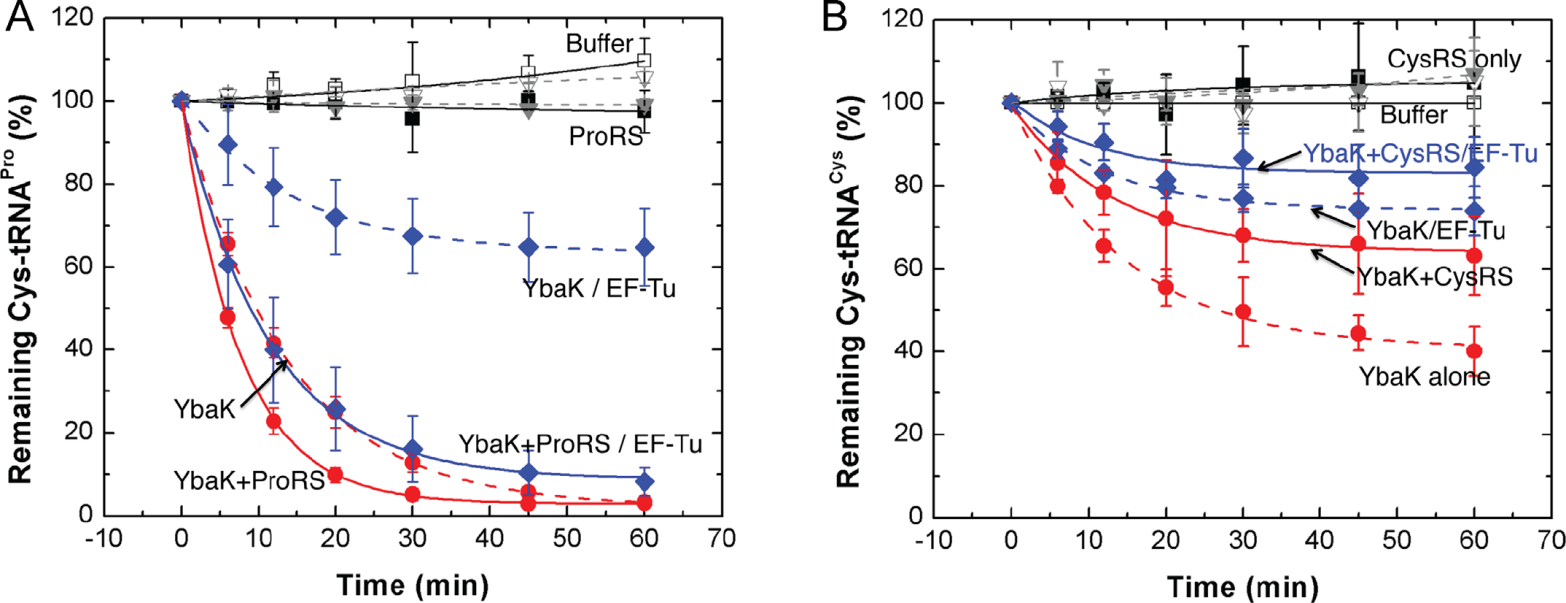
Cys-tRNA deacylation by YbaK or the YbaK/ARS complex in the absence and presence of EF-Tu. Deacylation reactions containing 0.5 μM Cys-tRNA, 0.1 μM YbaK, 0.5 μM ARS and 0.5 μM EF-Tu or BSA were carried out at 37°C. (**A**) Cys-tRNA^Pro^ deacylation by YbaK alone (red, dotted line), YbaK·ProRS (red, solid line), YbaK with EF-Tu (blue, dotted line), and YbaK·ProRS with EF-Tu (blue, solid line). (**B**) Cys-tRNA^Cys^ deacylation by YbaK alone (red, dotted line), YbaK·CysRS (red, solid line), YbaK with EF-Tu (blue, dotted line), and YbaK·CysRS with EF-Tu (blue, solid line). Controls with buffer or ARS alone are shown in black solid and dashed lines, respectively.

Previous studies showed that the presence of *E. coli* EF-Tu protects Cys-tRNA^Pro^ from deacylation by *H. influenzae* YbaK (23). We examined the effect of ProRS on Cys-tRNA^Pro^ deacylation by *E. coli* YbaK in the presence or absence of EF-Tu (Figure 8A). In the absence of EF-Tu, the ProRS/YbaK complex displayed only slightly enhanced deacylation activity compared to YbaK alone (Figure 8A, red solid and dashed lines, respectively). In the presence of EF-Tu, YbaK alone was able to deacylate Cys-tRNA^Pro^, but with considerably reduced efficiency (Figure 8A, blue dashed line), supporting the previous observation that EF-Tu can effectively compete with YbaK for substrate binding (23). Remarkably, addition of ProRS overcame the inhibitory effect of EF-Tu (Figure 6A, blue solid line), showing that interaction with ProRS enhances the ability of YbaK to deacylate Cys-tRNA^Pro^. We conclude that the ternary complex can more efficiently compete with EF-Tu for mischarged tRNA.

YbaK is a general Cys deacylase that deacylates both mischarged Cys-tRNA^Pro^ and correctly charged Cys-tRNA^Cys^ (22). We therefore examined the ability of YbaK to deacylate Cys-tRNA^Cys^ in the presence of CysRS and EF-Tu. In contrast to ProRS, the presence of CysRS reduced the rate of Cys-tRNA^Cys^ deacylation by YbaK (Figure 8B). Moreover, the presence of EF-Tu reduced deacylation further both in the absence and presence of CysRS. This suggests that both CysRS and EF-Tu protect Cys-tRNA^Cys^ from deacylation by YbaK. Collectively, these results support the specific effect of ProRS, but not CysRS, on YbaK deacylation of Cys-tRNA substrates.

## DISCUSSION

The recognition elements for aminoacylation by ARSs have been extensively investigated (39), whereas the editing determinants of tRNAs are less well understood (40–42). For some ARSs that possess editing capability, the identity elements for editing overlap with those for aminoacylation to some extent. For example, AlaRS recognizes the evolutionarily conserved wobble base pair G3:U70 on the acceptor stem of tRNA^Ala^, which is also crucial for editing by both the editing domain of AlaRS and the *trans*-editing factor AlaXp (41). Similarly, G34 of the anticodon is recognized by *E. coli* PheRS for both Phe-tRNA^Phe^ synthesis and Tyr-tRNA^Phe^ deacylation (33). The recognition elements important for aminoacylation by bacterial ProRS include anticodon bases G35/G36 and acceptor stem elements G72/A73, which are also important for the *cis* editing activity of ProRS and for the *trans*-editing protein ProXp-ala (38). In contrast, determinants for editing of *E. coli* ThrRS include residues in the D-loop of tRNA^Thr^, which do not affect aminoacylation (40). YbaK, which is the *trans*-editing factor that deacylates Cys-tRNA^Pro^, lacks inherent tRNA specificity (23,38). The mechanism of YbaK deacylation, thiol-specific cyclization of the substrate cysteine, supports the conclusion that it is a general Cys-tRNA deacylase (22,43). Previously, it has been proposed that a ternary complex of ProRS/tRNA^Pro^/YbaK confers YbaK tRNA specificity (23).

Here, we demonstrated the interaction between ProRS and YbaK in *E. coli* using split-GFP assembly. Since this assay was carried out in cells, it is possible that this interaction is mediated by tRNA^Pro^. AUC confirmed the binary interaction of ProRS/tRNA^Pro^, YbaK/tRNA^Pro^, and ProRS/YbaK pairs. Although we observed a YbaK/ProRS binary complex *in vitro*, we do not know if YbaK is found as a free-standing protein *in vivo* or always found in complex with ProRS or other synthetases. Additionally, AUC results are consistent with the existence of a ProRS/tRNA^Pro^/YbaK ternary complex. Furthermore, based on nanoESI IM-MS data, we conclude that the stoichiometry of the complexes is as follows: ProRS:tRNA^Pro^ (2:1), YbaK:tRNA^Pro^ (1:1), and ProRS:tRNA^Pro^:YbaK (2:1:1). This study represents the first ARS/tRNA/*trans*-editing factor complex observed without the need for crosslinking *in vitro*. We also performed competition studies using a high-resolution EMR mass spectrometer. This analysis allowed us to establish the specificity of complex formation in a mixture of cognate and non-cognate tRNAs. With EMR, different tRNAs can be distinguished based on small mass differences, allowing resolution of complexes containing different tRNAs without artificial tags. These experiments showed that YbaK formed complexes nonspecifically with various tRNAs, while ProRS and ProXp-ala bound cognate tRNA^Pro^ more specifically. Moreover, when ProRS, YbaK and three different tRNAs were mixed, a ternary complex was formed in significantly higher yield with cognate tRNA^Pro^ over the non-cognate tRNAs, suggesting that YbaK specificity for tRNA^Pro^ is mediated through interaction with ProRS.

In our study, a stoichiometry of two ProRS to one tRNA^Pro^ was consistently observed in both the two- and three-component complexes. This stoichiometry is in agreement with the crystal structure of *Thermus thermophilus* ProRS in complex with tRNA^Pro^, which displays asymmetry between the catalytic sites of ProRS dimers and shows only one tRNA^Pro^ molecule bound per dimer (44,45). A similar class II *T. thermophilus* SerRS dimer complexed with a single tRNA^Ser^ molecule was observed (46). Kinetic studies of histidyl-tRNA synthetase (HisRS) revealed that under single-turnover conditions only one AMP was formed per dimer of the enzyme (47). Moreover, a model for the class II ARS catalytic cycle was proposed based on rapid kinetic studies performed in the HisRS system (48). In this model, the first step is the formation of aminoacyl-adenylate in one of the two active sites, followed by binding of one tRNA to the monomer with the bound adenylate. Aminoacyl transfer then results in one aminoacyl-tRNA as the product, which stays bound to the dimer during amino acid activation, recruitment of another tRNA and aminoacyl transfer within the second active site of the dimer. A single tRNA molecule bound to a ProRS dimer, as detected in the native MS analysis, is consistent with an intermediate in this class II catalytic cycle model. Since only one tRNA is bound per ProRS dimer, the need for only a single YbaK protein is also reasonable. The lack of specificity of YbaK alone for a particular tRNA species (Figure 6) suggests that YbaK is not responsible for delivery of tRNA^Pro^ to the complex.

In conclusion, the finding that only a single YbaK protein binds to the 2:1 ProRS:tRNA complex, resulting in a 2:1:1 complex, supports the triple-sieve model for ProRS editing. The functional implications of ternary complex formation were also investigated by performing deacylation assays in the presence and absence of ProRS, CysRS, and EF-Tu. Taken together, these data support the conclusion that specific deacylation of Cys-tRNA^Pro^ is facilitated by interaction with ProRS, even in the presence of EF-Tu.

## Supplementary Material

Supplementary Data
