# Supplementary material for: Stoichiometry of triple-sieve tRNA editing complex ensures fidelity of aminoacyl-tRNA formation
Source: Nucleic Acids Res. 2018 Nov 12;47(2):929–40. doi: 10.1093/nar/gky1153 (PMC6344894; doi:10.1093/nar/gky1153)
Supplement: Supplementary Data [file gky1153_supplemental_files.pdf]

SUPPLEMENTARY DATA

Stoichiometry of triple-sieve tRNA editing complex ensures fidelity of aminoacyl-tRNA formation

Lin Chen[1,2,†], Akiko Tanimoto[1,†], Byung Ran So[1], Marina Bakhtina[1,2], Thomas J Magliery[1], Vicki Wysocki[1,2*] and Karin Musier-Forsyth[1,2,*]

[1] Department of Chemistry and Biochemistry, The Ohio State University, Columbus, OH, 43210, USA
[2] Center for RNA Biology, The Ohio State University, Columbus, OH, 43210, USA

* To whom correspondence should be addressed. Karin Musier-Forsyth, Tel: +1 614 292 202; Fax: +1 614 688 5402; Email: musier-forsyth.1@osu.edu. Correspondence may also be addressed to Vicki Wysocki, Tel: +1 614 292 8687; Fax: + 614 292 4628; Email: wysocki.11@osu.edu

† The authors wish it to be known that, in their opinion, the first two authors should be regarded as joint First Authors.
Present Address: [Byung Ran So], Department of Biochemistry and Biophysics, Howard Hughes Medical Institute and University of Pennsylvania School of Medicine, Philadelphia, PA, 19104, USA

SUPPLEMENTARY TEXT

Material and Methods

Materials

All amino acids and chemicals were purchased from Sigma-Aldrich unless otherwise noted. [α-^{32}P] ATP was from PerkinElmer Life Sciences.

Preparation of tRNA and aminoacyl-tRNA substrates

E. coli tRNA^{Pro} was prepared by *in vitro* transcription using T7 RNA polymerase (1) and 3'-[^{32}P]-end labeled using tRNA nucleotidyltransferase as previously described (2). Before the reactions, tRNA^{Pro} was refolded by heating at 80 °C for 2 min, 60 °C for 2 min, followed by addition of $MgCl_2$ to 10 mM and cooling to room temperature for 3 min. Ala-tRNA^{Pro} and Cys-tRNA^{Pro} were prepared by incubating 10 µM *C. crescentus* ProRS, 8 µM tRNA^{Pro} (trace amounts of ^{32}P-labeled tRNA mixed with 8 µM unlabeled tRNA), 60 mM Ala or Cys, 2 mM ATP and 0.02 mg/mL pyrophosphatase (Roche) for 5 min at room temperature in buffer containing 20 mM Tris-HCl, pH 8.0, 10 mM $MgCl_2$, 20 mM KCl, 0.2 mg/mL bovine serum albumin (BSA) and 10 mM dithiothreitol (DTT). After aminoacylation, mischarged tRNA substrates were phenol-chloroform extracted followed by ethanol precipitation and storage at -80 °C.

Aminoacylation assays

Each assay was performed in 50 mM HEPES, pH 7.5 and 20 mM KCl and also in buffer containing 50 mM ammonium acetate, pH 7.5 and 20 mM KCl. Aminoacylation assays were carried out at 37°C using 100 nM *C. crescentus* ProRS, 500 µM Pro, 6 µM ^{32}P-tRNA^{Pro} and 2 mM ATP in the presence of 10 mM $MgCl_2$, 0.2 mg/mL BSA and 10 mM DTT. Deacylation reactions were performed at 20°C in the presence of 5 mM $MgCl_2$, 0.1 mg/mL BSA, 2 mM DTT and 15 µg/mL inorganic pyrophosphate. Cys-tRNA^{Pro} deacylation was initiated by mixing with an equal volume of *C. crescentus* YbaK to obtain a final concentration of 0.9 µM Cys-tRNA^{Pro} and 0.5 µM YbaK. Ala-tRNA^{Pro} deacylation was initiated by mixing with an equal volume of *C. crescentus* ProXp-ala to a final concentration of 0.75 µM Ala-tRNA^{Pro} and 1 µM ProXp-ala. For each time point, reaction aliquots (2 µL) were quenched into 4 µL of 0.48 U/µL nuclease P1 in 200 mM NaOAc (pH 5). The reaction products (aa-[^{32}P]AMP and [^{32}P]AMP) were analyzed on polyethyleneimine-cellulose TLC plates as previously described (2).

MS simulation of binary complex of 2ProRS/tRNA and ternary complex of 2ProRS/tRNA/YbaK

The Gaussian function used is $f(x) = h \exp\left(\frac{-(x-(\frac{m+s+\sigma+z}{z}))^2}{\left(\frac{\sqrt{2}}{3z}\sigma\right)^2}\right)$, where x is *m/z*, m is the predicted mass of the target complex, σ is the peak width at the base, z is the charge state, h is the peak height, and *s* is mass shift. σ and *s* reflect the number of Mg^{2+} adducts that remain on complexes nonspecifically. These values are variable in each analysis due to slight differences in desolvation conditions caused by lack of uniformity of the shape and diameter of the capillary tips used for ionization. σ and *h* are arbitrarily determined by fitting to experimental peaks. *s* is determined from ProRS dimers observed within the same spectra. The variables used in the simulation are shown in Table S1.

REFERENCES

1. Beuning, P.J. and Musier-Forsyth, K. (2000) Hydrolytic editing by a class II aminoacyl-tRNA synthetase. Proc Natl Acad Sci U S A, 97, 8916-8920.
2. Ledoux, S. and Uhlenbeck, O.C. (2008) [3'-32P]-labeling tRNA with nucleotidyltransferase for assaying aminoacylation and peptide bond formation. Methods, 44, 74-80.

Supplementary Table S1

Variables used to simulate ProRS dimer, binary complex ProRS/tRNA, and ternary complex ProRS/YbaK/tRNAs. The values of mass shift and peak width are converted into number of Mg^{+2} and shown in parentheses.

				m	*σ*
concentrations	simulated spiecies	theoretical mass(Da)	mass shift (Da)	theoretical mass + mass shift (Da)	peak broadening (Da)
1 µM ProRS, 10 µM $tRNA^{Cys}$	observed ProRS dimer	98908	22(1)	98930	176(8)
	ProRS\$tRNA^{Cys}$	122910	66(3)	122976	264(12)
1 µM ProRS, 10 µM $tRNA^{Ala}$	observed ProRS dimer	98908	44(2)	98952	154(7)
	ProRS\$tRNA^{Ala}$	123591	88(4)	123679	242(11)
1 µM ProRS, 10 µM $tRNA^{Pro}$	observed ProRS dimer	98908	0(0)	98908	132(6)
	ProRS\$tRNA^{Pro}$	123754	44(2)	123798	220(10)
1 µM ProRS, 3.3 µM $tRNA^{Cys}$,3.3 µM $tRNA^{Ala}$,3.3 µM $tRNA^{Pro}$	observed ProRS dimer	98908	110(5)	99018	154(7)
	ProRS\$tRNA^{Cys}$	122910	154(7)	123064	242(11)
	ProRS\$tRNA^{Ala}$	123591	154(7)	123745	242(11)
	ProRS\$tRNA^{Pro}$	123754	154(7)	123908	242(11)
1 µM ProRS, 20 µM YbaK, 10 µM $tRNA^{Cys}$	observed ProRS dimer	98908	44(2)	98952	154(7)
	ProRS\$tRNA^{Cys}$	122910	88(4)	122998	242(11)
	ProRS\YbaK\$tRNA^{Cys}$	139559	132(6)	139691	242(11)
1 µM ProRS, 20 µM YbaK, 10 µM $tRNA^{Ala}$	observed ProRS dimer	98908	22(1)	98930	132(6)
	ProRS\$tRNA^{Ala}$	123591	66(3)	123657	220(10)
	ProRS\YbaK\$tRNA^{Ala}$	140241	110(5)	140351	220(10)
1 µM ProRS, 20 µM YbaK, 10 µM $tRNA^{Pro}$	observed ProRS dimer	98908	132(6)	99040	198(9)
	ProRS\$tRNA^{Pro}$	123754	176(8)	123930	286(13)
	ProRS\YbaK\$tRNA^{Pro}$	140403	220(10)	140623	286(13)
1 µM ProRS, 20 µM YbaK, 3.3 µM $tRNA^{Cys}$,3.3 µM $tRNA^{Ala}$,3.3 µM $tRNA^{Pro}$	observed ProRS dimer	98908	22(1)	98930	132(6)
	ProRS\$tRNA^{Cys}$	122910	66(3)	122976	220(10)
	ProRS\$tRNA^{Ala}$	123591	66(3)	123657	220(10)
	ProRS\$tRNA^{Pro}$	123754	66(3)	123820	220(10)
	ProRS\YbaK\$tRNA^{Cys}$	139559	110(5)	139669	220(10)
	ProRS\YbaK\$tRNA^{Ala}$	140241	110(5)	140351	220(10)
	ProRS\YbaK\$tRNA^{Pro}$	140403	110(5)	140513	220(10)

Supplementary Table S2

Sedimentation coefficient distribution of *C. crescentus* ProRS, *C. crescentus* YbaK, and *E. coli* $tRNA^{Pro}$ determined by sedimentation velocity analytical ultracentrifugation, as described in the main text.

Wavelength	679 nm	280 nm	256 nm
**AF680-YbaK**	1.82 ± 0.04	1.76 ± 0.01	1.77 ± 0.04
**ProRS**	N. A.	5.78 ± 0.07	5.76 ± 0.05
**$tRNA^{Pro}$**	N. A.	4.03 ± 0.03	4.02 ± 0.02

SUPPLEMENTARY FIGURES AND LEGENDS

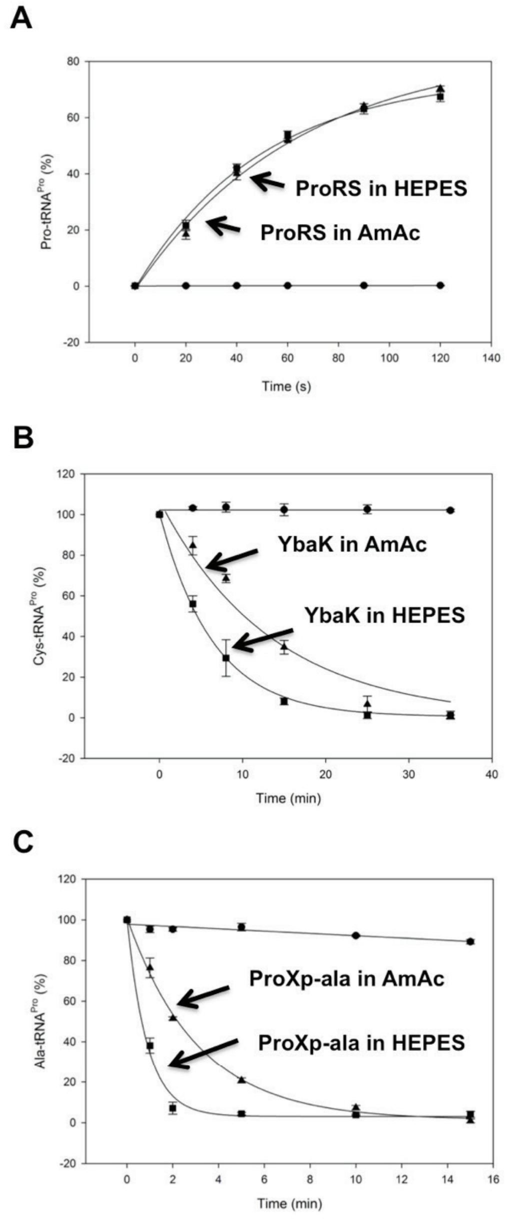

Supplementary Figure S1. Enzymatic activities of ProRS, YbaK, and ProXp-ala in HEPES buffer and ammonia acetate (AmAc) buffer.

A. Aminoacylation of 6 µM Pro-$tRNA^{Pro}$ by 100 nM ProRS in HEPES buffer (■), AmAc buffer (▲) and control with no enzyme (●). B. Deacylation of 0.9 µM Cys-$tRNA^{Pro}$ by 0.5 µM YbaK in HEPES buffer (■), AmAc buffer (▲) and control with no enzyme (●). C. Deacylation of 0.75 µM Ala- $tRNA^{Pro}$ by 1 µM ProXp-ala in HEPES buffer (■), AmAc buffer (▲) and control with no enzyme (●). All results are the average of three trials with the standard deviation indicated.

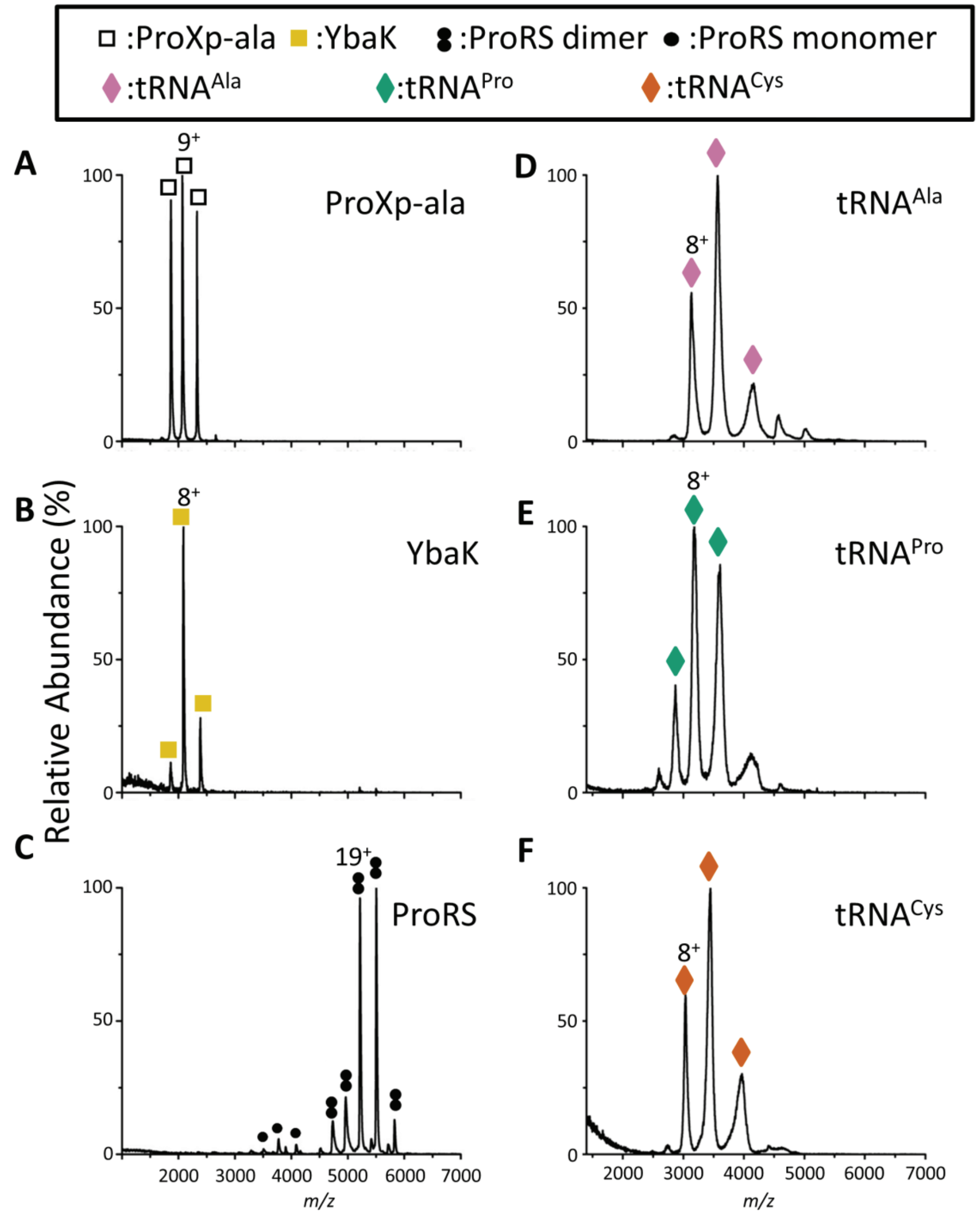

Supplementary Figure S2. Native mass spectra of individual components.

Full Spectra of (A) ProXp-ala, monomer (observed mass 18.6 kDa; theoretical 18.5 kDa); (B) YbaK, monomer (observed 16.7 kDa; theoretical 16.6 kDa); (C) ProRS, predominantly dimer (observed mass 99.2 kDa; theoretical 98.9 kDa); (D) $tRNA^{Ala}$, monomer (observed 25.0 kDa; theoretical 24.7 kDa); (E) $tRNA^{Pro}$, monomer (observed 25.5 kDa; theoretical 24.8 kDa); (F) $tRNA^{Cys}$; monomer (observed 24.1 kDa, theoretical 24.0 kDa).
